# Supplementary material for: Optimization of Rhodococcus erythropolis JCM3201T Nutrient Media to Improve Biomass, Lipid, and Carotenoid Yield Using Response Surface Methodology
Source: Microorganisms. 2023 Aug 24;11(9):2147. doi: 10.3390/microorganisms11092147 (PMC10534354; doi:10.3390/microorganisms11092147)
Supplement: Supplementary file 1 [file microorganisms-11-02147-s001.zip › microorganisms-2568089-supplementary.pdf]

Optimization of *Rhodococcus erythropolis* JCM3201^{T} nutrient media to improve biomass, lipid, and carotenoid yield using response surface methodology

Supplementary Materials

Table S1: Cell growth of *R. erythropolis* measured as OD at 600 nm over 192 h. Samples were cultured with variable nitrogen sources at elemental nitrogen concentration of 0.16 g L^{-1} (C:N of 100, 16 g L^{-1} carbon in the form of glucose), n = 3.

[h]	0	27	48	67	73	91	98	115	122	140	175	192
Ammonium chloride	0.2 ± 0	0.46 ± 0.01	0.79 ± 0.04	1.39 ± 0.07	1.42 ± 0.07	1.79 ± 0.12	2.06 ± 0.11	2.46 ± 0.14	2.45 ± 0.11	2.7 ± 0.09	3.65 ± 1.03	3.82 ± 0.08
Diammonium Hydrogen phosphate	0.2 ± 0	0.48 ± 0.02	0.83 ± 0.05	1.5 ± 0.09	1.53 ± 0.04	2.06 ± 0.11	2.18 ± 0.09	2.74 ± 0.04	2.75 ± 0.18	3.28 ± 0.43	4.26 ± 0.94	5.72 ± 0.83
Ammonium sulfate	0.2 ± 0	0.48 ± 0.02	0.86 ± 0.02	1.54 ± 0.15	1.59 ± 0.09	2.06 ± 0.07	2.24 ± 0.18	2.67 ± 0.07	2.77 ± 0.09	3.07 ± 0.22	4.16 ± 0.15	5.77 ± 0.78
Potassium nitrate	0.2 ± 0	0.42 ± 0.02	0.65 ± 0.03	0.81 ± 0.07	0.86 ± 0.05	1.16 ± 0.02	1.17 ± 0.06	1.25 ± 0.02	1.25 ± 0.05	1.35 ± 0.13	2.01 ± 0.11	1.93 ± 0.38
Ammonium nitrate	0.2 ± 0	0.48 ± 0.01	0.84 ± 0.04	1.48 ± 0.05	1.51 ± 0.18	1.98 ± 0.06	2.03 ± 0.08	2.55 ± 0.04	2.63 ± 0.11	3.42 ± 0.22	5.07 ± 0.19	5.76 ± 0.1
Yeast extract	0.2 ± 0	2.08 ± 0.08	3.55 ± 0.02	5.55 ± 0.11	6.3 ± 0.05	7.33 ± 0.28	7.66 ± 0.34	9.11 ± 0.19	8.64 ± 0.37	8.43 ± 0.22	9.47 ± 0.69	7.76 ± 0.49
Tryptone/Peptone	0.2 ± 0	1.66 ± 0.01	2.75 ± 0.08	4.69 ± 0.16	5.18 ± 0.09	6.36 ± 0.01	7.08 ± 0.19	7.5 ± 0.51	7.55 ± 0.02	7.72 ± 0.09	8.87 ± 0.59	7.96 ± 0.28
Urea	0.2 ± 0	0.49 ± 0.08	0.89 ± 0.09	1.43 ± 0.29	1.52 ± 0.31	2.28 ± 0.33	2.24 ± 0.41	2.76 ± 0.5	2.81 ± 0.57	3.57 ± 0.5	6.58 ± 0.26	6.78 ± 0.09
Ammonium acetate	0.2 ± 0	1.79 ± 0.03	2.9 ± 0.11	3.89 ± 0.22	4.64 ± 0.11	5.18 ± 0.29	5.83 ± 0.16	7.62 ± 0.13	7.46 ± 0.23	8.25 ± 0.26	10.52 ± 0.16	11.69 ± 0.37

Table S2: Biomass formation (DCW), lipid content (normalized to DCW) and Carotenoid accumulation (normalized to DCW) of *R. erythropolis* at 140 and 192 h. Samples were cultured with variable nitrogen sources at elemental nitrogen concentration of 0.16 g L^{-1} (C:N of 100, 16 g L^{-1} carbon in the form of glucose), n = 3.

	Biomass (g L^{-1})		Lipid content (mg g$^{-1}_{DCW}$)		Carotenoid content (Abs$_{454nm}$mg$^{-1}_{DCW}$)	
	140 h	192 h	140 h	192 h	140 h	192 h
Ammonium chloride	1.12 ± 0.11	1.66 ± 0.08	35.76 ± 13.79	48.68 ± 11.83	0.021 ± 0.003	0.015 ± 0.001
Diammonium hydrogen phosphate	0.99 ± 0.13	2.15 ± 0.14	40.12 ± 6.04	55.23 ± 0.64	0.039 ± 0.006	0.026 ± 0.006
Ammonium sulfate	1 ± 0.17	2.29 ± 0.08	44.76 ± 5.61	49.42 ± 0.21	0.035 ± 0.004	0.022 ± 0.005
Potassium nitrate	0.22 ± 0.12	0.36 ± 0.08	105.09 ± 25.09	79.27 ± 8.31	0.019 ± 0.006	0.008 ± 0.001
Ammonium nitrate	1.27 ± 0.16	1.72 ± 0.03	35.84 ± 3.09	49.62 ± 3.37	0.02 ± 0.004	0.014 ± 0
Yeast extract	2.13 ± 0.07	1.98 ± 0.08	103.85 ± 6.63	156.69 ± 22.99	0.026 ± 0.007	0.021 ± 0.006
Tryptone/Peptone	1.88 ± 0.03	1.72 ± 0.18	96.76 ± 2.66	132.29 ± 15.37	0.024 ± 0.006	0.021 ± 0.002
Urea	1.38 ± 0.19	2.3 ± 0.16	37.75 ± 4.86	46.46 ± 7.72	0.029 ± 0.002	0.015 ± 0.003
Ammonium acetate	2.58 ± 0.05	2.84 ± 0.02	51.13 ± 0.35	68.29 ± 4.98	0.021 ± 0.005	0.014 ± 0.002

Table S3: Cell growth of *R. erythropolis* measured as OD at 600 nm over 192 h. Samples were cultured with variable carbon sources at elemental carbon concentration of 16 g L^{-1} (C:N of 100, 0.16 g L^{-1} nitrogen in the form of ammonium acetate), n = 3.

[h]	0	18	24	41	48	67	94	111	121	140	164	192
Glucose	0.2 ± 0	0.96 ± 0.04	1.55 ± 0.18	2.47 ± 0.3	2.58 ± 0.33	3.21 ± 0.27	5.25 ± 0.29	6.25 ± 0.29	6.86 ± 0.32	8.22 ± 0.58	9.72 ± 0.56	9.81 ± 0.38
Galactose	0.2 ± 0	0.58 ± 0.1	0.84 ± 0.1	0.8 ± 0.2	0.66 ± 0.03	0.57 ± 0.1	0.8 ± 0.08	0.76 ± 0.09	0.75 ± 0.07	0.57 ± 0.11	0.88 ± 0.1	1.05 ± 0.17
Fructose	0.2 ± 0	3.07 ± 0.29	3.91 ± 0.33	4.61 ± 0.1	4.63 ± 0.18	3.85 ± 0.66	4.12 ± 0.49	3.31 ± 0.18	3.61 ± 0.49	3.47 ± 0.5	3.84 ± 0.62	3.79 ± 0.32
Lactose	0.2 ± 0	0.56 ± 0.07	0.63 ± 0.05	0.55 ± 0.04	0.59 ± 0.19	0.5 ± 0.08	0.66 ± 0.13	0.6 ± 0.09	0.64 ± 0.14	0.43 ± 0.06	0.63 ± 0.18	0.64 ± 0.13
Sucrose	0.2 ± 0	0.78 ± 0.06	1.41 ± 0.04	3.28 ± 0.3	3.5 ± 0.17	5.57 ± 0.4	8.37 ± 0.1	8.28 ± 0.36	8.39 ± 0.24	9.26 ± 0.31	9.68 ± 0.34	8.2 ± 0.29
Maltose	0.2 ± 0	0.79 ± 0.05	1.11 ± 0.13	1.45 ± 0.12	1.5 ± 0.34	1.25 ± 0.06	1.55 ± 0.11	1.42 ± 0.09	1.44 ± 0.06	1.48 ± 0.07	1.55 ± 0.09	1.4 ± 0.28
Sorbitol	0.2 ± 0	1.6 ± 0.03	2.38 ± 0.22	2.84 ± 0.16	2.96 ± 0.39	4.02 ± 0.31	5.4 ± 0.4	5.25 ± 0.19	5.62 ± 0.41	5.95 ± 0.5	6.48 ± 0.61	5.84 ± 0.57
Glycerol	0.2 ± 0	1.44 ± 0.04	1.94 ± 0.12	3.06 ± 0.19	3.28 ± 0.11	3.62 ± 0.36	4.56 ± 0.38	4.44 ± 0.41	4.56 ± 0.42	5.22 ± 0.18	5.31 ± 0.38	4.57 ± 0.05

Table S4: Biomass formation (DCW), lipid content (normalized to DCW) and Carotenoid accumulation (normalized to DCW) of *R. erythropolis* at 140 and 192 h. Samples were cultured with variable carbon sources at elemental carbon concentration of 16 g L^{-1} (C:N of 100, 0.16 g L^{-1} nitrogen in the form of ammonium acetate), n = 3. No carotenoid extraction was performed for galactose and lactose, due to a lack of sufficient biomass formation.

	Biomass (g L^{-1})		Lipid content (mg g^{-1}_{DCW})		Carotenoid content ($Abs_{454nm}mg^{-1}_{DCW}$)	
	140 h	192 h	140 h	192 h	140 h	192 h
Glucose	2.5 ± 0.13	3.1 ± 0.14	54.87 ± 2.86	75.9 ± 7.27	0.013 ± 0.001	0.015 ± 0.001
Galactose	0.24 ± 0.04	0.27 ± 0.02	24.52 ± 0.37	19.77 ± 0.43	-	-
Fructose	1.1 ± 0.12	1.35 ± 0.13	75.63 ± 8.48	73.94 ± 2.64	0.025 ± 0.001	0.021 ± 0.002
Lactose	0.25 ± 0.03	0.23 ± 0.04	38.5 ± 2.02	35.94 ± 5.11	-	-
Sucrose	2.69 ± 0.32	2.47 ± 0.22	52.63 ± 0.89	59.56 ± 0.9	0.017 ± 0.001	0.018 ± 0.001
Maltose	0.56 ± 0.08	0.43 ± 0.03	32.19 ± 2.47	34.95 ± 3.2	0.031 ± 0.011	0.027 ± 0.009
Sorbitol	1.79 ± 0.04	1.85 ± 0.14	81.84 ± 2.93	93.81 ± 4.11	0.018 ± 0	0.028 ± 0.008
Glycerol	1.46 ± 0.21	1.47 ± 0.06	65.49 ± 21.41	86.06 ± 13.43	0.028 ± 0.004	0.02 ± 0.001

Table S5: Estimated onset of stationary phase in *R. erythropolis* cultures cultivated in different nitrogen and carbon sources. Samples were cultured with at carbon concentration of 16 g L^{-1} and nitrogen concentration of 0.16 g L^{-1}, n = 3. For different nitrogen sources, glucose was used as carbon source. For different carbon sources, ammonium acetate was used as nitrogen source.

Nitrogen source	Onset of stationary phase [h]	Carbon source	Onset of stationary phase [h]
Ammonium chloride	-	Glucose	164
Diammonium hydrogen phosphate	-	Galactose	24
Ammonium sulfate	-	Fructose	48
Potassium nitrate	175	Lactose	24
Ammonium nitrate	-	Sucrose	94
Yeast extract	115	Maltose	48
Tryptone/Peptone	115	Sorbitol	94
Urea	-	Glycerol	94
Ammonium acetate	-		

Table S6: Fatty acid profiles of *R. erythropolis* at 140 and 192 h. Samples were cultured with variable nitrogen sources, n = 3. "Other" constitutes fatty acids with a representation below 3% of total fatty acid content (w/w), and include C14:1, C15:0, C17:0, 14-Methyl-C16:0, C18:2, C20:1, C20:3, C20:5, C22:1 among others.

**140 h**									
	**Ammonium chloride**	**Diammonium hydrogen phosphate**	**Ammonium sulfate**	**Potassium nitrate**	**Ammonium nitrate**	**Yeast extract**	**Tryptone/ Peptone**	**Urea**	**Ammonium acetate**
**C14:0**	0.163 ± 0.01	0.16 ± 0.011	0.161 ± 0.013	0.162 ± 0.017	0.156 ± 0.002	0.117 ± 0.003	0.117 ± 0.002	0.146 ± 0.007	0.123 ± 0.003
**C16:0**	0.254 ± 0.003	0.247 ± 0.005	0.253 ± 0.004	0.271 ± 0.01	0.266 ± 0.004	0.336 ± 0.001	0.337 ± 0.003	0.235 ± 0.004	0.304 ± 0.003
**C16:1**	0.118 ± 0.003	0.12 ± 0.003	0.118 ± 0.004	0.1 ± 0.009	0.107 ± 0.004	0.05 ± 0.001	0.054 ± 0.001	0.117 ± 0.003	0.067 ± 0.001
**C17:1**	0.078 ± 0.004	0.076 ± 0	0.073 ± 0.003	0.065 ± 0.006	0.069 ± 0.003	0.034 ± 0.001	0.034 ± 0	0.071 ± 0	0.047 ± 0.001
**C18:0**	0.004 ± 0.006	0 ± 0	0 ± 0	0.014 ± 0.003	0.01 ± 0	0.071 ± 0.003	0.066 ± 0.002	0.006 ± 0.005	0.032 ± 0.002
**C18:1 (oleat)**	0.092 ± 0.005	0.093 ± 0.003	0.093 ± 0.001	0.099 ± 0.006	0.095 ± 0.001	0.164 ± 0.008	0.162 ± 0.003	0.097 ± 0.001	0.133 ± 0.003
**C18:3**	0.004 ± 0.007	0.009 ± 0.008	0.008 ± 0.007	0.024 ± 0.003	0.012 ± 0.003	0.016 ± 0	0.016 ± 0.001	0.019 ± 0.005	0.035 ± 0.002
**10-Me-C18:0**	0.26 ± 0.013	0.262 ± 0.005	0.265 ± 0.004	0.243 ± 0.015	0.256 ± 0.008	0.125 ± 0.002	0.142 ± 0.002	0.249 ± 0.006	0.2 ± 0.004
**Other**	0.027 ± 0.023	0.034 ± 0.018	0.029 ± 0.017	0.02 ± 0.007	0.028 ± 0.012	0.087 ± 0.007	0.073 ± 0.003	0.06 ± 0.005	0.059 ± 0.002
**192 h**									
	**Ammonium chloride**	**Diammonium hydrogen phosphate**	**Ammonium sulfate**	**Potassium nitrate**	**Ammonium nitrate**	**Yeast extract**	**Tryptone/ Peptone**	**Urea**	**Ammonium acetate**
**C14:0**	0.134 ± 0.023	0.136 ± 0.005	0.132 ± 0.009	0.146 ± 0.019	0.134 ± 0.003	0.103 ± 0.001	0.11 ± 0.005	0.138 ± 0.015	0.109 ± 0.003
**C16:0**	0.309 ± 0.002	0.3 ± 0.007	0.307 ± 0.002	0.265 ± 0.004	0.279 ± 0.002	0.341 ± 0.013	0.341 ± 0.008	0.301 ± 0.018	0.317 ± 0.003
**C16:1**	0.067 ± 0.002	0.07 ± 0.004	0.068 ± 0.001	0.096 ± 0.005	0.096 ± 0.001	0.044 ± 0.002	0.047 ± 0.001	0.072 ± 0.003	0.056 ± 0.001
**C17:1**	0.05 ± 0.006	0.05 ± 0.001	0.05 ± 0.003	0.068 ± 0.003	0.063 ± 0.001	0.03 ± 0.001	0.031 ± 0	0.051 ± 0.004	0.039 ± 0
**C18:0**	0.027 ± 0.004	0.027 ± 0.004	0.028 ± 0.003	0.016 ± 0.002	0.017 ± 0.001	0.08 ± 0.009	0.078 ± 0.002	0.023 ± 0.006	0.051 ± 0.003
**C18:1 (oleat)**	0.11 ± 0.011	0.118 ± 0.002	0.117 ± 0.006	0.107 ± 0.005	0.109 ± 0.003	0.182 ± 0.014	0.18 ± 0.002	0.117 ± 0.012	0.164 ± 0.006
**C18:3**	0.018 ± 0.003	0.026 ± 0.004	0.025 ± 0.004	0.025 ± 0.007	0.022 ± 0.001	0.002 ± 0.001	0.007 ± 0.007	0.032 ± 0.006	0.004 ± 0
**10-Me-C18:0**	0.206 ± 0.013	0.203 ± 0.004	0.208 ± 0.003	0.239 ± 0.005	0.245 ± 0.003	0.115 ± 0.001	0.122 ± 0.001	0.206 ± 0.008	0.163 ± 0.005
**Other**	0.078 ± 0.013	0.07 ± 0.017	0.065 ± 0.007	0.039 ± 0.009	0.036 ± 0.004	0.104 ± 0.035	0.084 ± 0.002	0.061 ± 0.003	0.095 ± 0.011

Table S7: Fatty acid profiles of *R. erythropolis* at 140 and 192 h. Samples were cultured with variable carbon sources, n = 3 (except for Maltose-192 h, where n = 2 as one sample was excluded as an outlier). "Other" constitutes fatty acids with a representation below 3% of total fatty acid content (w/w), and include C14:1, C17:0, 14-Methyl-C16:0, C18:3, C20:1, C20:3, C20:5, C22:1 among others.

**140 h**								
	**Glucose**	**Galactose**	**Fructose**	**Lactose**	**Sucrose**	**Maltose**	**Sorbitol**	**Glycerol**
**C14:0**	0.137 ± 0.001	0.125 ± 0.022	0.074 ± 0.002	0.089 ± 0.022	0.144 ± 0.006	0.106 ± 0.013	0.123 ± 0	0.103 ± 0.004
**C15:0**	0.012 ± 0.003	0.009 ± 0.008	0.011 ± 0.001	0.011 ± 0.01	0.011 ± 0.001	0 ± 0	0.02 ± 0.002	0.031 ± 0.003
**C16:0**	0.306 ± 0.01	0.304 ± 0.024	0.32 ± 0.012	0.275 ± 0.01	0.307 ± 0.002	0.258 ± 0.005	0.318 ± 0.001	0.326 ± 0.01
**C16:1**	0.068 ± 0.003	0.112 ± 0.012	0.091 ± 0.001	0.128 ± 0.008	0.082 ± 0.001	0.132 ± 0.002	0.074 ± 0	0.084 ± 0.023
**C17:1**	0.045 ± 0.003	0.059 ± 0.012	0.023 ± 0	0.062 ± 0.002	0.051 ± 0	0.088 ± 0.004	0.037 ± 0.001	0.019 ± 0.002
**C18:0**	0.034 ± 0.004	0 ± 0	0.048 ± 0.004	0 ± 0	0.028 ± 0.001	0 ± 0	0.047 ± 0.001	0.044 ± 0.017
**C18:1 (oleat)**	0.125 ± 0.002	0.051 ± 0.009	0.199 ± 0.009	0.035 ± 0.003	0.084 ± 0.001	0.012 ± 0.011	0.129 ± 0.002	0.181 ± 0.023
**10-Me-18:0**	0.198 ± 0.011	0.279 ± 0.028	0.143 ± 0.005	0.372 ± 0.019	0.231 ± 0.003	0.378 ± 0.003	0.179 ± 0.002	0.152 ± 0.037
**Other**	0.075 ± 0	0.06 ± 0.042	0.091 ± 0.007	0.027 ± 0.004	0.06 ± 0.001	0.026 ± 0.001	0.074 ± 0.002	0.06 ± 0.013

	192 h							
	**Glucose**	**Galactose**	**Fructose**	**Lactose**	**Sucrose**	**Maltose**	**Sorbitol**	**Glycerol**
**C14:0**	0.115 ± 0.004	0.131 ± 0.041	0.071 ± 0.001	0.082 ± 0.01	0.142 ± 0.005	0.115 ± 0.011	0.105 ± 0.001	0.103 ± 0.005
**C15:0**	0.019 ± 0.003	0.014 ± 0.004	0.011 ± 0	0.017 ± 0.002	0.012 ± 0	0.006 ± 0.008	0.024 ± 0.002	0.031 ± 0.004
**C16:0**	0.32 ± 0.009	0.353 ± 0.026	0.326 ± 0.007	0.277 ± 0.014	0.304 ± 0.004	0.241 ± 0.005	0.311 ± 0.002	0.33 ± 0.007
**C16:1**	0.055 ± 0.002	0.096 ± 0.041	0.089 ± 0.01	0.126 ± 0.001	0.081 ± 0.001	0.123 ± 0.013	0.077 ± 0.001	0.063 ± 0.009
**C17:1**	0.037 ± 0.001	0.047 ± 0.012	0.023 ± 0.001	0.064 ± 0.005	0.055 ± 0	0.093 ± 0.007	0.041 ± 0	0.019 ± 0.002
**C18:0**	0.053 ± 0.003	0.022 ± 0.038	0.045 ± 0.002	0 ± 0	0.033 ± 0.003	0.007 ± 0.01	0.05 ± 0.002	0.052 ± 0.008
**C18:1 (oleat)**	0.151 ± 0.006	0.078 ± 0.066	0.209 ± 0.004	0.031 ± 0.006	0.08 ± 0.001	0.03 ± 0.029	0.131 ± 0.002	0.206 ± 0.023
**10-Me-18:0**	0.157 ± 0.011	0.219 ± 0.042	0.142 ± 0.001	0.381 ± 0.015	0.222 ± 0	0.332 ± 0.068	0.178 ± 0.002	0.119 ± 0.016
**Other**	0.092 ± 0.027	0.04 ± 0.01	0.084 ± 0.009	0.022 ± 0.014	0.072 ± 0.002	0.052 ± 0.074	0.084 ± 0.002	0.077 ± 0.003

Table S8: ANOVA of central composite design (CCD) models after 192 h.

**Biomass 192 h**						
**Source**	**Sum of Squares**	**df**	**Mean Square**	**F-value**	**p-value**	
**Model**	84.07	3	28.02	131.83	< 0.0001	significant
A-Carbon	5.55	1	5.55	26.13	0.0001	
B-Nitrogen	75.75	1	75.75	356.38	< 0.0001	
AB	2.76	1	2.76	12.99	0.0024	
**Residual**	3.4	16	0.2126			
Lack of Fit	1.9	5	0.3806	2.79	0.0722	not significant
Pure Error	1.5	11	0.1362			
**Cor Total**	87.47	19				
**Std. Dev.**	0.4611		**R^2**	0.9611		
**Mean**	4.25		**Adjusted R^2**	0.9538		
**C.V. %**	10.85		**Predicted R^2**	0.9416		
			**Adeq Precision**	33.0434		
**Lipid content 192 h**						
**Source**	**Sum of Squares**	**df**	**Mean Square**	**F-value**	**p-value**	
**Model**	6333.18	3	2111.06	36.5	< 0.0001	significant
A-Carbon	357.83	1	357.83	6.19	0.0243	
B-Nitrogen	4889.89	1	4889.89	84.54	< 0.0001	
B^2	1879.87	1	1879.87	32.5	< 0.0001	
**Residual**	925.42	16	57.84			
Lack of Fit	889.71	5	177.94	54.82	< 0.0001	significant
Pure Error	35.71	11	3.25			
**Cor Total**	7258.59	19				
**Std. Dev.**	7.61		**R^2**	0.8725		
**Mean**	36.13		**Adjusted R^2**	0.8486		
**C.V. %**	21.05		**Predicted R^2**	0.7262		
			**Adeq Precision**	18.9127		
**Carotenoide content 192 h**						
**Source**	**Sum of Squares**	**df**	**Mean Square**	**F-value**	**p-value**	
**Model**	0.0001	3	0	20.51	< 0.0001	significant
A-Carbon	0	1	0	15.74	0.0011	
B-Nitrogen	0	1	0	41.87	< 0.0001	
B^2	9.94E-06	1	9.94E-06	8.72	0.0094	
**Residual**	0	16	1.14E-06			
Lack of Fit	7.55E-06	5	1.51E-06	1.55	0.2525	not significant
Pure Error	0	11	9.73E-07			
**Cor Total**	0.0001	19				
**Std. Dev.**	0.0011		**R^2**	0.7936		
**Mean**	0.0173		**Adjusted R^2**	0.7549		
**C.V. %**	6.17		**Predicted R^2**	0.6715		
			**Adeq Precision**	13.7974		
